# Epitaxial growth of crystal phase quantum dots in III–V semiconductor nanowires

**DOI:** 10.1039/d2na00956k

**Published:** 2023-03-06

**Authors:** Miguel Sinusia Lozano, Víctor J. Gómez

**Affiliations:** a Nanophotonics Technology Center, Universitat Politècnica de València, Camino de Vera s/n Building 8F, 2a Floor 46022 Valencia Spain vjgomher@ntc.upv.es

## Abstract

Crystal phase quantum dots (QDs) are formed during the axial growth of III–V semiconductor nanowires (NWs) by stacking different crystal phases of the same material. In III–V semiconductor NWs, both zinc blende (ZB) and wurtzite (WZ) crystal phases can coexist. The band structure difference between both crystal phases can lead to quantum confinement. Thanks to the precise control in III–V semiconductor NW growth conditions and the deep knowledge on the epitaxial growth mechanisms, it is nowadays possible to control, down to the atomic level, the switching between crystal phases in NWs forming the so-called crystal phase NW-based QDs (NWQDs). The shape and size of the NW bridge the gap between QDs and the macroscopic world. This review is focused on crystal phase NWQDs based on III–V NWs obtained by the bottom-up vapor–liquid–solid (VLS) method and their optical and electronic properties. Crystal phase switching can be achieved in the axial direction. In contrast, in the core/shell growth, the difference in surface energies between different polytypes can enable selective shell growth. One reason for the very intense research in this field is motivated by their excellent optical and electronic properties both appealing for applications in nanophotonics and quantum technologies.

## Abstract 2

This review summarizes the epitaxial growth of crystal phase quantum dots in nanowires in terms of basic concepts, control of the crystal phase in the axial direction, shell growth in the radial direction and optical and electronic properties.

## Graphical abstract

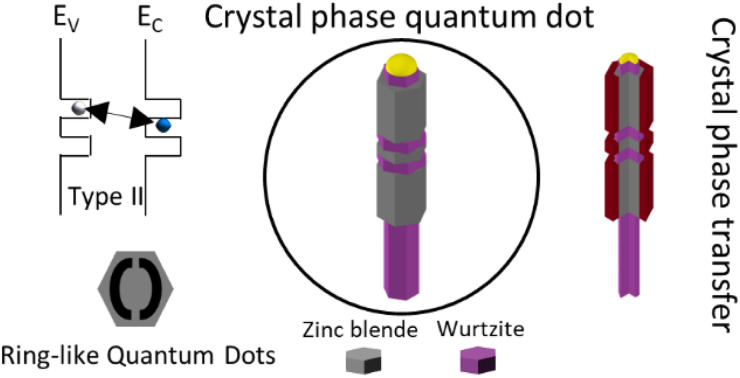

## 1. Introduction

This review is focused on crystal phase QDs formed in III–V semiconductor NWs by stacking, in the axial direction, WZ or ZB crystal phases of the same material by the bottom-up VLS growth method. General concepts of WZ and ZB polytypes will first be introduced. Then, the growth mechanism and control over the nucleation, layer growth, and crystal phase selection will be reviewed together with the process of selective core/shell growth. Following this, the review will cover the optical and electronic properties including specific examples and case studies.

Nanoscale structures, such as NWs, enable heterostructures to overcome the limitations related to lattice mismatch and strain relief, whereas the growth of their planar counterparts is strongly constrained to a small lattice parameter window, therefore, offering additional degrees of freedom, for example in terms of materials combination, for the formation of low dimensional heterostructures, including quantum wells (QW), quantum wires (QWRs), and QDs embedded in NWs, also known as nanowire quantum dots (NWQDs). The three-dimensional carrier confinement and localized/discrete carrier states in QDs are predicted^1^ to introduce new physical phenomena to diode lasers and other photonic devices. Traditionally NWQDs can be produced *via* heterostructured interfaces between materials with different band gaps (*i.e.* switching materials and/or alloy composition) or by changing the crystal structure without changing the material. Epitaxially grown NWs with embedded QDs offer a way for fully deterministic positioning of the QDs. Moreover, the shape and aspect ratio of the NWs guarantee the link of the QD with the macroscopic world, either by electrical contacts or photon emission.

The combination of NWs and QDs is crucial for photonics due to the enhanced spontaneous emission rate by coupling generated light with cavity modes (Purcell effect), waveguiding, and suppression of the total internal reflection so that the efficiency of light extraction can theoretically approach 100%.^2–6^ Additionally, the directionality of emissions can be controlled by engineering the shape of the tip of the NW,.^7^ thus resulting in the realization of novel nanophotonic devices based on nanowires. In agreement with this, NWQD-based single-photon subwavelength detectors^8,9^ and emitters^10,11^ have been demonstrated. NWQDs can also easily be addressed *via* electrical contacts. Therefore, QDs can be electrically pumped when embedded in an NW axial p–n junction. This allows the injection of electron–hole pairs and their subsequent radiative recombination into photons emitted by the QD.^12^ In addition, virtual QDs can be induced by localized electrical gating, allowing a versatile manipulation of the density of states in the QD.^13,14^ In this case, the QD-like confinement is achieved by local tuning of the Fermi level by applying the right voltage by means of precisely positioned gate electrodes instead of engineering the QD by a local difference either in material, composition or crystal phase.

Two different approaches can be distinguished for the fabrication of NWQDs: top-down and bottom-up.

### 1.1 Top-down fabrication of NWQDs

The top-down approach involves the breaking down of a substrate including QDs into nanostructures such us NWs. By using this fabrication method precisely positioned one-dimensional structures can be obtained. Depending on the density of QDs in the substrate and the NW diameter (defined *via* lithography), the resulting NWQDs can embed one or several QDs.^15–17^ One of the main drawbacks of this approach is the impact of etching on exposed surfaces, also referred to as etch damage, hindering the material quality and the subsequent device performance. Moreover, as this approach needs a substrate containing QDs, this technique is limited to materials and material combinations which already exist in bulk, which is not generally the case for different crystal phases.

In general, self-assembled semiconductor QDs are grown in the Stranski–Krastanov (SK) growth mode. The SK growth mode is driven by the lattice mismatch between the substrate and the growing layer. After growing a few monolayers (wetting layer), the accumulated strain is relieved *via* the random nucleation and growth of island-like formation (QDs).^18^ The resulting QDs can then be embedded in the material after the epitaxial growth of a new epi-layer called the capping layer. The optical properties of the QDs depend on the composition (in the case of ternary or quaternary alloys), size, and strain. It is worth highlighting that the composition of the alloy defines the lattice parameter, which is related to the strain and, therefore, to the size of the QDs. Moreover, the emission energy of the QDs is directly related to the surrounding material (wetting and capping layers), determining the charge carrier confinement in the QD. One of the main drawbacks of self-assembled QDs is the lack of uniformity in heights and lateral size, ranging typically between 1–10 and 10–70 nm respectively.^19^ The density and size distribution can be controlled up to some extent by varying the growth conditions.^20^ Another disadvantage is the material inter-mixing between the QD and capping layer that can take place during the growth, which further increases the variation in the optical properties of the QDs.^21^ Therefore, the top-down approach must deal with QDs ensembles with limited control in location, size, density, and material uniformity.

### 1.2 Bottom-up fabrication of NWQDs

However, in the bottom-up approach chemical or physical forces operating at the nanoscale during the growth process are employed to assemble the NWs where the QDs are included. This can be achieved by changing the growth conditions, precursor species or precursor flows. The bottom-up approach offers a way to grow NWQDs with smooth sidewalls with axial and/or radial heterostructures or doping profiles. Due to the nanowire geometry, lattice mismatch and the resulting stress can be accommodated *via* strain relaxation in the radial direction without the introduction of defects, *i.e.*, dislocations. This allows us to obtain NWQDs in both the axial and radial directions.^22–24^ An additional advantage of NWQDs to be considered is their relatively high surface-to-volume ratio compared to their planar counterparts that allows the existence of other crystal phases not stable in the bulk (polytypism).^25–27^ In III–V NWs, ZB and WZ alternating segments can be successfully controlled to confine charges and obtain type-II NWQDs within the same material.^28–31^

**Fig. 1:**
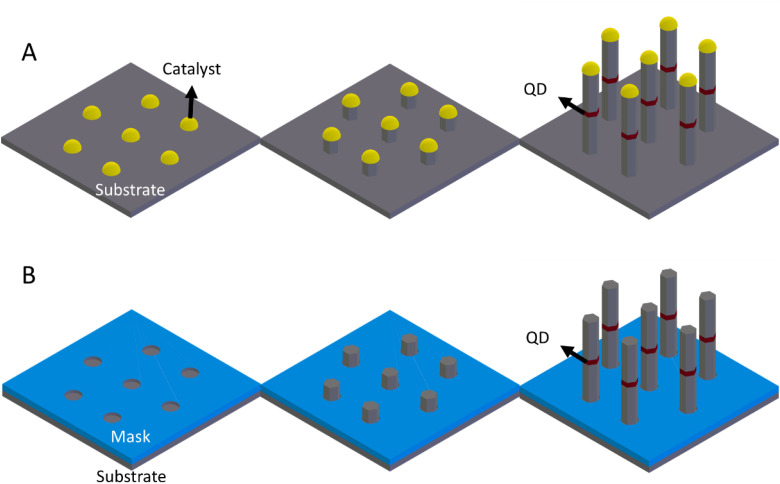
Schematic representation of the NW growth modes. (A) VLS growth mode where the axial growth is catalysed by a metallic droplet (typically Au). (B) SAE growth mode where the growth takes place at an opening in a mask layer.

The bottom-up NW growth approach relies either on the VLS (Fig. 1A) or on the selective-area epitaxy (SAE, Fig. 1B). It is worth mentioning that the main difference between the two growth modes is in the axial direction, being the radial growth dominated by the vapor–solid growth mode which is similar in both cases. Both growth modes, VLS or SAE, allow forming NWQDs. Compared to the top-down fabrication, there are some major advantages when using the bottom-up growth of NWQDs: a wider material compatibility, deterministic fabrication down to a single QD per NW, and natural alignment of the QD along the NW axis.

The VLS NW growth mode,^32^ originally introduced by Wagner and Ellis in 1964,^33^ is mediated by a metallic droplet in the liquid phase, which collects the growth species introduced in the reactor chamber in the vapor phase, leading to the nucleation of the crystal in the solid phase, below the droplet. Then, the growing NW lifts the droplet that remains at the tip of the NW. In 1991, Hiruma and co-workers^34^ grew, for the first time, III–V compound nanowires. One year later, in 1992, the same group reported the co-existence of both ZB and WZ crystal structures in indium arsenide NWs.^35^ In 1994, Murayama and co-workers reported the calculations of the band offsets at WZ–ZB interfaces.^36^ However, it was not until 2008 that the first successful attempts to control the crystal structure during NW growth^37,38^ and the first optical studies^39–42^ were reported. This progress practically enables engineering of the electronic structure of a single material. For instance, a WZ/ZB/WZ structure can define a quantum well in a single material. Growing such a structure in a NW will also confine charges in two other dimensions, forming a crystal phase NWQD. During the past few decades, a huge effort has been made in order to understand the VLS growth process, thus leading to NWs with high crystal quality,^43^ controlled crystal phase,^27,44^ controlled axial doping,^45^ and to obtain axial and radial heterostructures.^22,46,47^ Despite the significant progress made, the detailed growth mechanism is not fully understood yet and research in this area is going on.

The metallic droplet material can either be one of the group-III growth species (leading to a self-catalysed or self-assisted growth) or a foreign material, being Au the most widespread and studied.^48^ The metallic droplets, catalysts, can be randomly deposited on the substrate by means of spark discharge,^49^ aerosol generation,^50^ or annealing of Au thin films.^51^ Additionally, the NW growth sites can be lithographically defined showing the upscaling possibilities of this technology.^52–55^

Since metal contamination from the metallic droplet in the NWQD could be detrimental to its optical and electrical properties, self-catalysed or catalyst-free growth methods have been developed.^56–58^ Also in this case, arrays of NWs can be grown on a lithographically patterned substrate.^58^ The SAE is a catalyst-free direct vapor–solid growth and is based on growth mask layers with openings that define the NW growth. The growth is driven by differences in the nucleation and crystal growth on different crystal facets mainly due to differences in surface energies.^59^ The openings in the growth mask expose the underlying substrate and define the locations where the epitaxy of the NWs starts. For the axial growth along the 〈111〉 direction, the conditions are chosen such that the {110} facets have significantly lower growth rates than the {111} facets. Then, the NW grows with {110} lateral facets and the NW shows a hexagonal cross-section. Under the appropriate conditions, the radial growth can be fully suppressed, and the NW diameter remains equal to the mask opening. Fig. 1B shows the schematic representation of the SAE growth.

As with VLS growth, it is possible to obtain high crystal quality,^60^ control the crystal phase,^61^ switch between axial and radial growth,^62^ dope the NW during growth,^62^ and obtain axial and radial heterostructures.^62,63^ In principle, SAE growth is less complex than VLS growth, as only two phases, vapor and solid, are involved. Nevertheless, the growth dynamics are highly complex, for example the dependence on the diameter of the mask openings and distance between openings, on the NW geometry, and on the relative supply of group III (or V) constituents in the growth of ternaries.^64–66^ Therefore, the main challenges in the SAE growth method are to obtain a high degree of control in NW growth and in understanding its subtleties.

In general, NWs and NWQDs can be grown by different techniques ranging from metal–organic vapor-phase epitaxy (MOVPE),^2,28,67^ to chemical beam epitaxy (CBE),^68^ and molecular beam epitaxy (MBE).^56,69^ Moreover, NWs and NWQDs can be *in situ* characterized by transmission electron microscopy during growth by either MOVPE^70,71^ or MBE.^57^ Furthermore, crystal phase heterostructures can be exploited not only with NWs, but also with nano-membranes^72,73^ and microdisks.^74^ The area of NWQDs is extremely broad with application examples in several fields such as quantum technologies^75,76^ and nanophotonics.^77,78^

## 2. Crystal phase axial NWQDs

The idea behind the growth of NWQDs is relatively simple: a material with a lower band gap should be inserted inside another material with a higher band gap. Thus, forming an axial structure of the style A/B/A, where B shows quantum confinement due to the differences in band gap energy and the relative position of the band edges. The stacking sequence is obtained typically by changing the growth precursors during the axial NW growth. The patterning of the catalysts in VLS controls the position of the nanowire, and the diameter of the catalyst defines the NW and QD diameters. The axial position of the QD and its length are defined by the growth time. Therefore, the combination of patterning together with the VLS bottom-up growth offer full control over the size and position of the QD inside the NW and on the substrate. Moreover, radial growth can be employed either to fully embed the QD inside the NW or to grow an oxide shell, using atomic layer deposition (ALD), to improve the waveguiding properties of the NW.^79^ Another advantage of axial NWQDs arises when the NW diameter is properly chosen. This way, the emission from the QD can be efficiently coupled to the fundamental waveguiding mode of the NW, thus enhancing the extraction efficiency. Nowadays, this is possible thanks to a deep understanding and control of the growth process. In addition, axial NWQDs can be electrically injected when contacting the region above and below the QD. In this case, electrons, and holes flow from the top region to the bottom one going through the QD region defined in between.

### 2.1 Material composition *vs.* crystal phase QDs

The axial growth of III–V NWQDs relies on the possibility to change the material composition or crystal phase during growth. Two approaches can be differentiated to realize NWQDs. First, by heterostructuring semiconductor materials with different band gaps, or second, by tuning the crystal phase of a single material. For the first approach, the perfection of the NWQD is limited by the difficulty to control the interface abruptness and material composition at the atomic level^80–82^ which unfortunately are determining factors for the physical properties of QDs as they define the shape of the potential well.^29,83^ Recent studies have shown that perfect atomically sharp interfaces can be achieved if the crystal structure is changed instead of the material composition^28,29,84^ and the crystal structure can be controlled using basic growth parameters^43,44^ creating crystal phase QDs, which, in some aspects, supersede material QDs. As the interfaces are defined between different crystal phases in the same material, no atom intermixing can occur, creating atomically sharp WZ–ZB interfaces.^28,85^ Moreover, there is minimal strain at a WZ–ZB interface.^86^ This makes polytype heterostructures ideal candidates for the exploration of the low dimensionality properties of NWQDs. In addition, single electron transport^83^ and quantum light sources have been demonstrated using crystal phase NWQDs.^87^

Regardless of the specific growth technique employed (MBE, MOCVD, Au-assisted, self-catalysed…), the way to define a crystal phase NWQD depends on the band alignment between WZ and ZB phases, and this is specific to a particular material. For example, in the case of InP, GaAs, and InAs there is a type-II band alignment between WZ and ZB,^88^ thus leading to the confinement of electrons in the ZB phase when it is sandwiched between WZ barriers. Moreover, these type-II structures can support the formation of indirect excitons at the interfaces between crystal phases (Fig. 4). Finally, the main limitation of the crystal phase approach is the lack of flexibility in the formation of QDs and the fixed and relatively small band offsets. Moreover, there are materials where it is not possible to form crystal phase QDs in a controlled way. This is the case of III-nitrides.

### 2.2 Crystal phases: zinc blende and wurtzite

**Fig. 2:**
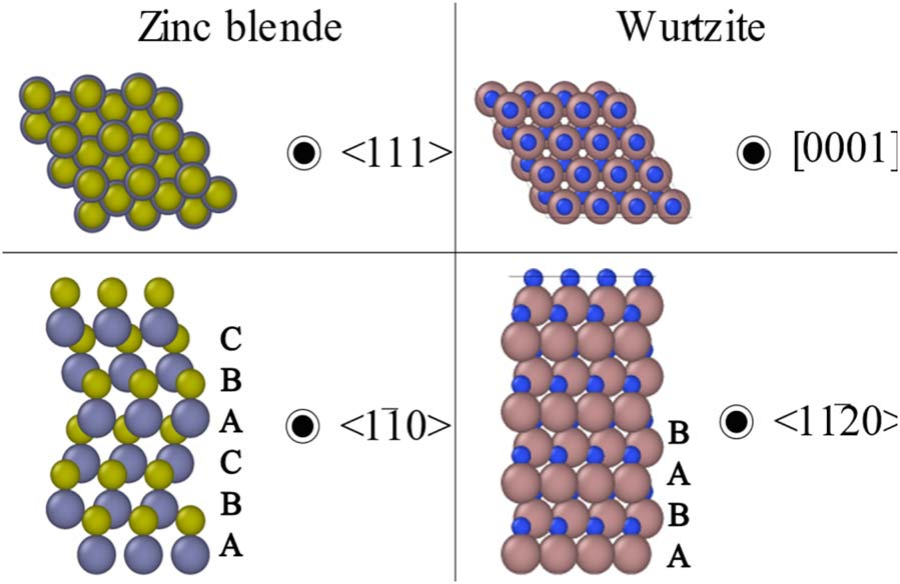
The atomic arrangement for the ZB and WZ structures.

Before describing the axial growth of crystal phase NWQDs, it is necessary to fully describe the crystal structures to be investigated: ZB and WZ. Both crystal phases differ only in their stacking sequence in the close-packed directions (〈111〉 in ZB and 〈0001〉 in WZ), as is shown in the top part of Fig. 2. Both structures consist of hexagonally close-packed planes of III–V pairs. The difference lies in the alignment of these hexagonally close-packed planes with their surrounding planes. For ZB, three sequential layers form a repeating unit (…ABCABCABC…), where each letter represents one of the three allowed positions of the pairs of group-III and group-V atomic layers along the specific 〈111〉 direction. For the WZ crystal phase, only two of the allowed positions are occupied, giving the stacking sequence …ABABAB… Otherwise, each atomic layer can be denoted separately, differentiating the two different atomic species, using lower-case and upper-case letters. Then the ZB and WZ stacking sequences can be written as …AaBbCcAaBbCc… and …AaBbAaBb…, respectively (see the bottom part of Fig. 2). When there is an intermixing of crystal structures and/or different faults appear in their stacking sequence, this notation becomes mandatory to fully describe and distinguish between different defects and stacking faults in the sequence of these two types of crystal structures.^89^

**Fig. 3:**
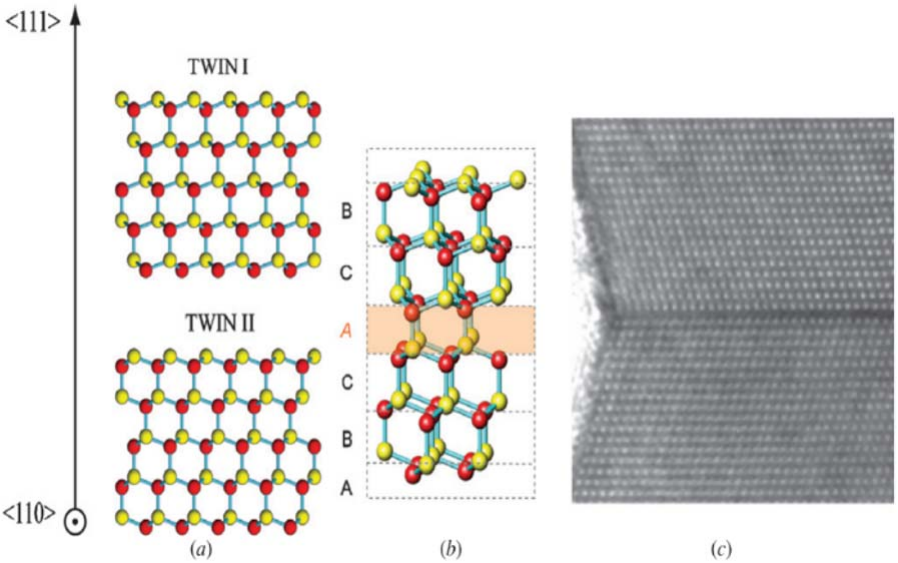
(a) Two different twin orientations of the ZB stacking sequence, denoted as ‘I’ and ‘II’. (b) When the two twin orientations of ZB are combined, the common plane A is a twin plane or twin plane. (c) Transmission electron microscopy image of a section of an InAs nanowire exhibiting two different orientations of zinc blende stacking (twin segments), separated by a twin plane. In the TEM image, group III and V atoms in adjacent planes cannot be resolved and appear as a single dot. Fig. 2 from Kimberly A Dick *et al.*, 2010, *Semicond. Sci. Technol.*, **25**, 024009, reproduced with the permission of IOP Publishing.

In III–V semiconductor NWs, the most frequently reported defects are twin planes, stacking faults, and the lack of control over the WZ and ZB formation. For the discussion on how to control the crystal phase, the reader is referred to Section 2.4.2. In general, twin planes and stacking fault defects have a negative impact on the optical and electronic properties of III–V semiconductor NWs by reducing the carrier lifetime, mobility, and quantum efficiency.^90^ On the other hand, stacking faults can have a positive impact on some of the physical properties of NWs as in the case of the field emission properties of GaN NWs.^91,92^ It has also been reported that there is an increase in Young's modulus with the increase in stacking faults for GaAs NWs.^93^ Twin planes are commonly observed in the ZB crystal phase. There, the stacking sequence is often found to unexpectedly change from an …ABCABCABC… stacking sequence to an …ABCACBACBA… The stacking sequence still corresponds to the ZB-type sequence after ‘A’, but before and after this bilayer the sequences are mirrored in ‘A’. This ‘mirror’ plane (A) is known as a twin plane and the sequences above and below the mirror plane have different twin orientations (Fig. 3). Nevertheless, the small part of the sequence surrounding the twin plane (…ABCACBACBA…) can be regarded as a WZ-type stacking sequence. In this review, we will follow the criteria published by Dick and co-workers^89^ where they exclusively use the terms twin and twin plane for this type of defect or interruption in the stacking sequence. They use WZ only when the WZ-type stacking sequence happens over at least four bilayers. Moreover, the term stacking faults refer to interruptions in the WZ stacking sequence such as …ABABCBABA… where C is the erroneous bilayer creating the stacking fault. Different strategies have been implemented to successfully reduce the density of stacking faults. Controlling the growth temperature and V/III ratio,^38,43^ NW diameter^94^ or the use of growth interruption strategies^95^ has been demonstrated to be critical in reducing the density of stacking defects.

The different stacking sequences for ZB and WZ slightly change the local environment for each atom in the crystal lattice. In particular, the third-nearest-neighbour spacing is shorter in WZ. The shift in cohesive energy ranges from around −45 meV per III–V pair for AlN^96^ to around 25 meV for GaSb.^97^ This difference in bulk cohesive energy between the two phases is the reason why WZ is the preferred crystal structure for III-nitrides and, for example, WZ GaAs is not stable in bulk. Moreover, ZB and WZ crystal structures also have different surface terminations, as shown on the right-hand side of Fig. 2. In particular, for NWs growing in the 〈111〉/〈0001〉 directions, the surfaces present in the sidewalls of the NWs are different for the two structures.^98,99^ Although there is a wide dispersion in the estimates of what the actual energies of these surfaces are, most of the reports agree that the WZ {1010} surface should have a lower surface energy than the ZB {110}.^98–101^

### 2.3 The role of supersaturation

Supersaturation is a state of thermodynamic instability and hence the system becomes prone to reduce the supersaturation through the process called precipitation. Therefore, supersaturation is the driving force of the epitaxial growth process. Supersaturation can be defined as the difference in chemical potential between the growth environment and the substrate.

1 Δ*μ* = *μ*_gas_ − *μ*_subs_

At equilibrium, there is no difference between the chemical potential, the growth environment, and the chemical potential of the substrate. Therefore, Δ*μ* = 0. To achieve crystal growth, a supersaturated growth environment is required. Crystal growth continues as long as the chemical potential of the growth environment is higher than that of the substrate. When the chemical potential of the substrate is higher than that of the growing environment, then the etching of the substrate is promoted over crystal growth.

For NWs in the VLS mode, growth occurs at the interface between the catalyst and NW, so this discussion will be focused on the chemical potential difference between the interface and the supply atoms. Supersaturation is affected by the growth temperature, pressure, and concentration of the growth species at the catalyst–NW interface. The concentration of growth species is directly controlled by the partial pressures of the precursors. However, it is vital to highlight that supersaturation is affected by many other growth parameters. As mentioned before, it depends on growth temperature and pressure. In general, the chemical potential difference between a vapour and its solid phase will decrease with temperature. However, for growth techniques that involve molecular precursors, such as MOVPE, the precursor decomposition rate depends exponentially on temperature, which increases the supersaturation for fixed precursor molar fractions. Moreover, surface reconstruction of the substrate varies with temperature and vapour molar fractions and directly affects the surface diffusion of adatoms. Therefore, the dependence of the supersaturation on the growth parameters is highly complex. NW systems are even more complicated. For example, when growth is diffusion limited, *i.e.*, when the density of the NWs is high compared to the supply material, NWs compete for the supply material and the supersaturation per nanowire is therefore reduced. Supersaturation decreases as the NW grows away from the substrate, reducing the amount of material reaching the metallic particle, as reported for InP NWs.^102^ Other factors may include the local variation in precursor decomposition rate, solubility of the seed particle (which can be varied with temperature), trace impurities, and dopants in the system (which may affect adsorption, diffusion, and dissolution kinetics).

For III–V semiconductors, the group III and V elements, two different species, must combine to form the growing NW. These two species follow different, not always independent, kinetic processes. Both species have different adsorption, diffusion, and desorption rates, affecting their individual supersaturations at the catalyst–NW interface. When molecular precursor species are used, another degree of complexity is added to the system. This is because the two different precursor molecules will have to decompose first to deliver the atomic species to the growth front at a different decomposition rate. In addition, precursor molecules may compete for reactants, or react with each other at the surface or in the gas phase generating undesired by-products. Determining the optimum ratio of precursor molar fractions required to obtain equal adatom concentrations of the two species at the catalyst–NW interface is not straightforward. Therefore, the molar fraction ratio between the two species (V/III ratio) is an important parameter for the optimum NW growth.

### 2.4 Nanowire growth

**Fig. 4:**
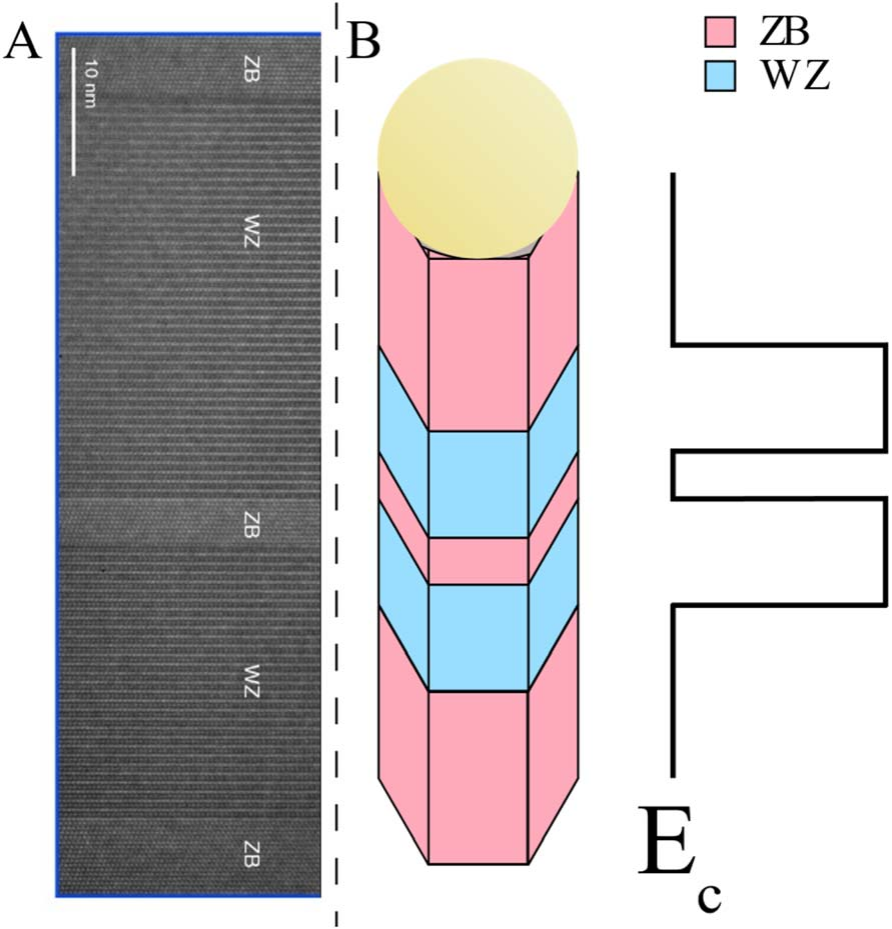
(A) High-resolution transmission electron micrograph of an InAs NW with a ZB QD sandwiched between WZ barriers. (B) Schematic representation of the crystal structure stacking sequence and the resulting type-II band alignment. Adapted from Fig. 1 by Potts, H., Chen, I., Tsintzis, A. *et al.*, Electrical control of spins and giant *g*-factors in ring-like coupled quantum dots, *Nat. Commun.*, **10**, 5740, (2019), https://doi.org/10.1038/s41467-019-13583-7 Is licensed under CC BY 4.0.

The crystal phase sequence in the area surrounding the QD is of the style A/B/A, where crystal phase B shows quantum confinement. In the VLS method, the material forming the NW is dissolved in the metal droplet and, when reaching supersaturation, precipitates at the droplet–NW (liquid–solid) interface. Layer by layer, generating the longitudinal growth of the NW. However, multilayer growth has also been demonstrated.^103^ After a certain growth time and for a chosen NW length, the growth conditions are changed to switch to crystal phase B. Note that the growth rate may change. At the desired length of the segment of crystal phase B, the growth conditions are changed to switch back to crystal phase A. Eventually, the growth is stopped, and the result is a NW of a single material containing a crystal phase QD of the style A/B/A. Fig. 4 illustrates an example of crystal phase NWQD in InAs of the style WZ/ZB/WZ. If the dimensions of the lower band gap crystal phase are small enough, the corresponding segment should show quantum confinement and behave like a QD.

After that, the growth conditions can be switched to promote radial growth and a shell can be added to finalize the NWQD structure. This can be useful to create 3D structures by growing a shell (or a selectively grown shell) of a second material that replicates the crystal phase of the core.^22,104,105^ Despite III–V semiconductor NWs typically growing vertically, stochastic processes of growth parameter modulation can lead to a change in the growth direction. This is the kinking phenomenon. For a deeper insight into the kink-type defects, the reader is kindly referred to the review published in 2022 by Vlassov and co-workers.^106^

#### 2.4.1 Controlling the nucleation and layer growth

In the VLS growth method, the material forming the NW is dissolved in the metal droplet and, when reaching supersaturation, precipitates at the droplet–NW (liquid–solid) interface, thus, generating the longitudinal growth of the NW layer by layer. Controlling this process is critical to developing nanomaterials with atomic precision, and therefore, to meet the demands of nanophotonics, electronics, and quantum technology applications. During the last decade, *in situ* experiments have been performed and the NW growth process has been recorded. This has allowed scientists to solve questions regarding growth dynamics.^71,107–109^ In the growth process of a new layer two steps can be differentiated: the incubation and the layer growth across the liquid–solid interface.^110,111^ As the catalyst size is relatively small, the number of atoms needed to reach supersaturation is small, so they can be consumed during the growth of a layer.^112^ Then, the catalyst droplet needs to accumulate enough material to reach the supersaturation state again and to overcome the nucleation barrier for a new layer. Therefore, the incubation time can be defined as the time interval between the end of the growth of a layer and the beginning of the following one.^71,107,113^ This interpretation implies that there is a nucleation step during which the critical nucleus forms. In general, this nucleation step is extremely fast and not visible in experiments. After the incubation time, the droplet reaches supersaturation again and a new layer is formed. Therefore, the layer completion time can be defined as the time observed for the completion of each layer. In the literature, the layer completion time is also referred to as the step-flow time.

In 2020 Maliakkal and co-workers^114^ reported the independent control of nucleation and layer growth in Au-catalysed GaAs NWs using *in situ* TEM imaging. They demonstrated that the time scales for the nucleation and layer growth are of similar magnitude but follow different trends. According to their findings, the incubation time can be controlled by tuning the Ga precursor partial pressure, while the layer growth time can be controlled using the flow of the As precursor. They observed a similar order of magnitude for the incubation and layer completion times. The layer completion times range from 0.1 to 0.5 s, and the incubation times range from about 0.2 to 5 s. The underlying reason of why Ga and As control the growth in a different way is the ease with which Ga alloys with the metallic catalyst while the As incorporation into the catalyst is comparatively low. Their findings can be generalized for binary materials where each of the species interacts very differently with the catalyst. Therefore, offering a mechanism to independently control the nucleation and layer growth. On the one hand, the incubation step is controlled by the atomic species that easily alloys with the catalyst droplet, given the fact that every atom increases the chemical potential of the alloy by small steps. On the other hand, the layer growth is controlled by the other atomic species, that, in comparison, its dissolution into the droplet is much smaller. Therefore, for the other atomic species only very few atoms are required to supersaturate the catalyst droplet.

In contrast with the previous discussion, multilayer growth has been observed for binary compound NWs.^108,115^ Moreover, when growing ternary NWs, a third element (group III or V) is incorporated. It is worth mentioning that the layer evolution in ternary NWs can determine and affect the resulting composition.^116^ Recently, Sjökvist and co-workers reported the multilayer growth of InGaAs NWs.^104^ They observed that the fluctuations in the flow of growth species in and out the metal nanoparticle influence the composition of the growing NW.

#### 2.4.2 Controlling the crystal phase

The control over the NW crystal structure has been achieved by several research groups *via* the variation of different process parameters: (i) growth temperature; (ii) radius of the NW; (iii) introduction of foreign species; and (iv) nominal V/III-ratio.

##### 2.4.2.1 Growth temperature

The growth temperature has been successfully employed for controlling the crystal phase.^38,43,55,117^ However, using the temperature for controlling the crystal phase increase significantly the complexity of the process. The timespan for changing the temperature generally ranges between minutes and tenths of minutes, therefore making it extremely challenging, or even impossible, to form sharp interfaces and short segments and thus minimizing two of the main advantages of crystal phase QDs. Moreover, temperature changes affect almost all relevant growth parameters^118^ such as chemical potentials, precursor pyrolysis, migration length, effective V/III-ratio, and the total amount of available material, therefore making temperature changes really complicate to be considered theoretically.

##### 2.4.2.2 Radius of the NW

Apart from growth temperature, the radius of growing NWs plays a key role in the selection between the ZB and the WZ crystal structure. From an empirical perspective, the radius of the NW is well known to affect the crystal structure selection^26,94,119^ but from a theoretical point of view the role of the nanowire radius is still under debate. Johansson and co-workers^119^ presented a theoretical model that explained the observed WZ preference in thin nanowires in terms of the Gibbs–Thomson (GT) effect. The GT effect refers to a reduction of the supersaturation, the driving force for growth when the size of nanowires decreases.^120^ These results differ from the ones reported by Dubrovskii and co-workers^121,122^ that consider both, the solid and liquid phases when calculating the GT effect. They concluded that the GT effect does not have an influence in the choice of crystal structure. Different thermodynamic stability studies have reported that the WZ structure can become energetically favourable for a sufficiently small NW radius (with transition radii ranging from a few to a few tens of nanometres).^99,123–125^ However, pure ZB NWs have been grown with a radius of 5 nm.^126^ Therefore, in terms of crystal phase selection, the connection between theory and experiment is limited.

Recently, Mårtensson and co-workers developed a stochastic simulation model^127^ where the main input parameters are the flows of the growth species and the temperature of the process, allowing a direct transference of results between the simulations and the experimental epitaxial process. They use the model to simulate GaAs NW growth with varying As flows, obtaining a good agreement between their simulation results and the trends observed experimentally.

In a later paper,^128^ they applied the developed model to analyse the effect of the radius on the crystal structure selection of GaAs nanowires. To understand the potential effects of the NW radius, they simulated the growth at different As flows and expanded the model to account for the GT effect and the surface diffusion length of Ga atoms. First, the GT effect results in a size-dependent effective supersaturation due to the change in the surface area of the seed particle upon nucleation. Second, the incorporation of Ga atoms can occur *via* surface diffusion and direct impingement, which gives rise to a radius-dependent growth rate.^122^ They concluded that the geometry of the system (*i.e.* radius of the NW) plays a key role in the dynamics of the seed particle composition throughout the growth. This directly controls the supersaturation, which is of vital importance for VLS NW growth. As the radius of the NW decreases, nucleation takes place at higher values of supersaturation, thus promoting the formation of the WZ crystal structure.^110,122^ Moreover, when in addition to direct impingement, considering the group-III atoms that reach the seed particle by means of diffusion, the V/III ratio becomes radius dependent. Therefore, NWs with smaller radii typically grow with lower V/III ratios, and the V/III ratio is one of the parameters controlling the crystal phase.

##### 2.4.2.3 Introduction of foreign species

The term foreign species is employed here to refer to species that are not strictly needed for growth. For example, in GaAs, we are considering foreign species to all other materials different from the Ga or As precursor. The introduction of dopants^37,129–131^ or gases, such as HCl,^132^ has been employed to control the crystal structure of MOVPE-grown NWs but the effect of foreign species on precursors is rather complex. Hence, it cannot be excluded that several growth parameters are indirectly affected as well. Furthermore, with respect to device fabrication, doping, and diameter should remain free variables rather than a necessary parameter for controlling the crystal phase.

##### 2.4.2.4 Nominal V/III-ratio

Control of crystal structure has also been reported by varying the nominal V/III ratio, and the total precursor flows.^27,44,117,133–135^ In 2013, Lehmann and co-workers^44^ reported crystal phase control in GaP, GaAs, InP, and InAs by tuning only the group-V precursor flow, while keeping constant all other growth parameters, promoting the bidirectional transition from WZ to ZB. They successfully fabricated sharp WZ–ZB and ZB–WZ interfaces. This is a simple yet powerful approach to control the polytypic behaviour of Au-seeded GaP, GaAs, InP, and InAs NWs by only tuning one growth parameter, the group-V precursor flow. They found that WZ is promoted when the group-V precursor flow value is about 50–150 times larger than the corresponding ZB. In contrast to temperature variations, this approach allows for fast (in the order of a few seconds) switching between ZB and WZ. It also leaves other process parameters (particle diameter and doping) as degrees of freedom.

**Fig. 5:**
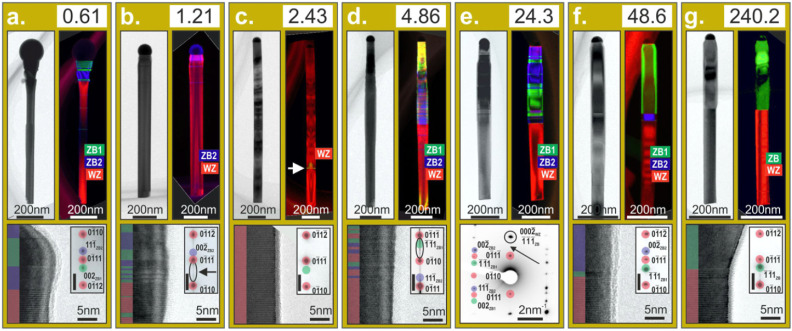
(a–g) Detailed TEM analysis of dual GaAs nanowires grown with a WZ stem and lower, similar, and higher nominal V/III ratios for the top segment with the respective V/III-ratio given at the top right of each figure. The subfigures in each panel include a TEM bright field image, a colour-coded TEM dark field composite image (red-WZ, green-ZB twin 1, blue-ZB twin 2), a high-resolution TEM image of the interface area, and a SADP with the colour-coding on the pattern as used for the dark field images. The scale bar in the SADP insets is 1 nm^−1^. Fig. 3 from Sebastian Lehmann *et al.*, 2015, *Nanotechnology*, **26**, 301001, reproduced with the permission of IOP Publishing.

However, the early studies in crystal structure control showed opposite tendencies for increasing group-V supply in molecular beam epitaxy (MBE) and metalorganic vapour phase epitaxy (MOVPE). More specifically, in MBE, the WZ crystal phase is promoted for increasing group-V supply in Au^110,136–138^ and self-catalysed growth^139–141^ while ZB is favoured in MOVPE for GaAs,^27,85^ InAs,^27,94,117,133^ InP,^44,142^ and GaP.^95,111,143–145^ However, few MBE studies reported similar tendencies as in MOVPE for Au-seeded NW growth.^146,147^ In 2015, Lehmann and co-workers reported^135^ that the growth regimes (nominal V/III ratio) for crystal phase control in MBE and MOVPE are not mutually exclusive. By increasing the V/III ratio (changing only group-V precursor flow) in Au-catalysed GaAs NW growth it is possible to switch from a regime favourable to the ZB-to-WZ transition, to another regime where the WZ-to-ZB transition is favourable. They grew a set of NWs with increasing V/III-ratio from 0.61 until 240.2 (Fig. 5). All NWs were initiated with a stem grown at a nominal V/III ratio of 2.43 (conditions previously found to yield the WZ structure),^44^ followed by a second step were only the group-V precursor flow, and consequently, the V/III ratio was varied. They observed that ZB is formed for nominal V/III ratios well below (Fig. 5a) and above (Fig. 5g) the reference value of 2.43 for WZ (Fig. 5c). Nevertheless, twin-free ZB was only formed for a nominal V/III ratio of 240.2 (Fig. 5g). For intermediate V/III ratios a mixed crystal structure (WZ together with both ZB twin orientations) is promoted (Fig. 5b, d–f). A clear transition from WZ to ZB was observed for nominal V/III ratios away from the reference for WZ (2.43). Therefore, ZB occurs for decreasing the V/III ratio in the lower range for increasing the V/III ratio in the higher range. In addition, they showed that the first transition occurs for a V-limited growth regime, while the second transition arises in a III-limited regime, with WZ occurring when the group-III and group-V precursors are balanced.

Similar results were observed for the crystal phase control in InSb NWs. The effect of group-V and group-III flows on morphology and crystal structure in InSb NW was investigated by Anandan and co-workers.^134^ They observed that the growth of InSb NWs in group-V limited and/or rich regime promoted the ZB crystal structure, while WZ mixed with twinned ZB is promoted when group-V and group-III precursors are effectively balanced. Besides, the WZ formation window is investigated by precisely controlling the group-V flow.

### 2.5 Selective core/shell growth

The difference in surface energies between the WZ and ZB polytypes enables, under the appropriate growth conditions, a crystal phase selective core/shell growth. The selective shell growth opens new opportunities for the design of NW devices. This selective growth is demonstrated for different core/shell combinations, such as GaAs/InAs,^148,149^ InAs/GaSb,^104,150^ and InAs/AlSb.^151^ In the work of Gómez and co-workers,^148^ they identify the two key factors governing the core/shell selectivity in GaAs/InAs core/shell NWs: shell growth rate and shell growth time. They attributed the occurrence of the selectivity to either a difference in crystal phase or a difference in lateral facet termination. Selectivity was found for short growth times and high growth rates. However, for long enough growth times the selectivity was lost, and homogeneous shells were grown, thus, limiting the maximum thickness of the shell growth under selective conditions. To overcome this limitation, they demonstrated a two-step growth formed by a high growth rate step followed by a low growth rate. By doing so, they demonstrate thickness control while keeping the shell selective growth.

Another opportunity offered by the core/shell growth is the realization of crystal phases which otherwise would be impossible to obtain by means of the crystal structure transfer method because the shell replicates the crystal structure of the core. This way a material in a non-stable crystal phase can be stabilized when growing on the sidewalls of a crystal structure-controlled NW. For example, the lonsdaleite crystal structure of Si has been demonstrated when growing on WZ GaP NWs.^152^ Another example is the realization of WZ GaSb on InAs crystal structure-controlled NWs. Generally, in III–V NWs the WZ crystal structure is relatively easy to form. This is not the case for III–Sb NWs, where obtaining the WZ crystal structure is highly challenging. The reasons behind this behaviour can be found in the low ionicity of the III–Sb bond and in the surfactant effect of Sb and its effects on the surface energy of the system.^153^ Those limitations can be overcome when growing a III–Sb shell on a crystal-structure engineered InAs NW under the appropriate growth conditions. By properly tuning the growth conditions, it is possible to realize WZ AlSb^151^ or WZ GaSb.^150^

## 3. Properties of NWQDs

In this section, we give a brief overview on the properties of axial bottom-up crystal phase NWQDs. We are going to focus on their optical and electronic properties.

### 3.1 Optical properties

In general, the WZ–ZB interfaces in III–V semiconductors have a type-II (staggered) band alignment.^88^ A type-II band alignment occurs when, on one side of the junction both, the conduction and valence bands are shifted in the same direction (upwards or downwards) with respect to the other side of the junction. The relative position of the lowest conduction band should be above (in energy) the highest valence band. Otherwise, one should talk about type-III (broken gap band alignment). It is worth highlighting again that the main advantages related to the crystal phase NWQDs are that perfect atomically sharp interfaces can be achieved^28,29,84^ and the absence of material intermixing.^80–82^

In GaAs, at low temperatures, the energy of the WZ and ZB band gaps is very close.^31,154^ The theoretical calculations of the band offset between WZ-GaAs and ZB-GaAs range from 70 to 150 meV,^36,88,155,156^ while they have been experimentally measured between 50 and 120 meV.^31,155^ Typically, the band offset for a type-II band alignment is determined across the type-II interface using the energy (in the photoluminescence, PL, spectrum) of the spatially indirect recombination. In InP, the band gap of the WZ crystal phase is 1.49 eV at low temperatures,^157–159^ 70 meV higher than the band gap of the ZB phase, and is shifted to higher energy by the valence band offset.^88^ Theoretical calculations give a difference ranging from 45 to 80 meV^36,88,156^ with experimental values measured between 35 and 70 meV.^160^ In the WZ–ZB–InP interface it is possible to create a 2DEG free to move in the radial but confined in the axial direction.^160^ For a deeper insight into the band offsets between WZ and ZB in III–V semiconductors, the reader is kindly referred to the work done by Belabbes^88^ and Luiz H. Galvão Tizei^161^ and co-workers.

**Fig. 6:**
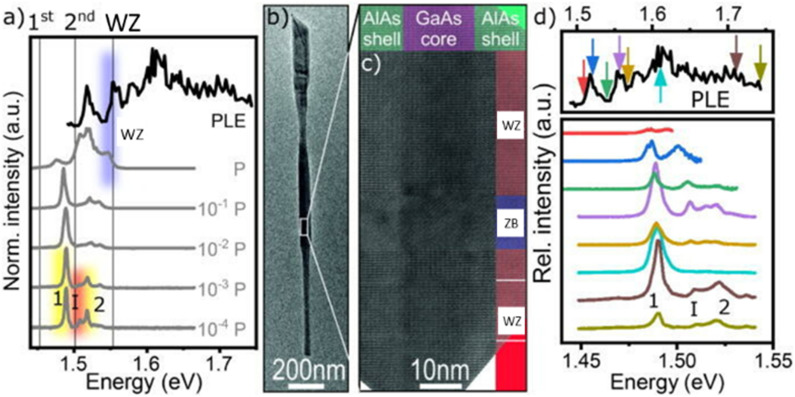
(a) The normalized PLE (black) and power-dependent PL spectra with varying EPD over four orders of magnitude (grey). The emission from the WZ-GaAs band gap, the first and second Q-dot states, and the WZ–ZB GaAs interface are labelled WZ (blue), 1 and 2 (yellow), and I (red), respectively. The calculated transition energies for the first and second Q-dot states, as well as the WZ-GaAs band-to-band emission, are indicated with black lines. (b) Bright-field TEM image of the nanowire. (c) High-resolution TEM image of the ZB-GaAs dot. (d) PL spectra of the first and second Q-dot states and the interface states acquired at different excitation energies. An arrow of identical color in the included PLE spectrum indicates the excitation energy for each spectrum. Fig. 3 from Geijselaers. I, *et al.*, *Appl. Phys. Lett.*, **119**, 263102, (2021); https://doi.org/10.1063/5.0072151, reproduced with the permission of AIP Publishing.

An example of the excellent properties that can be obtained is illustrated by the work done by Geijselaers and co-workers in 2021.^28^ They fabricated GaAs NWs with individual crystal phase QDs (Fig. 6). The diameters of the NWs ranged between 15 and 25 nm, thus resulting in radial confinement of charge carriers. They included a 20 nm thick AlAs passivation layer covered with a 5 nm AlGaAs shell to prevent oxidation. They employed TEM for the structural characterization and photoluminescence spectroscopy (PL) and photoluminescence excitation spectroscopy (PLE) to confirm a sharp density of states (DOS) in the QDs.

The normalized power-dependent PL spectra (grey) of a NW containing a ZB-GaAs QD are shown in Fig. 6a. The QD inspected had a diameter of 16.6 nm and a length of 13 nm, as demonstrated by TEM (Fig. 6b and c). For the highest excitation power densities, the spectrum is dominated by the WZ-GaAs band-to-band emission at 1.547 eV. This indicates a radial confinement of about 30 meV, comparable with previous reports with similar NWs.^162^ The PL signal of the WZ band-to-band emission vanishes at low excitation power densities. Three individual peaks, labelled 1, I, and 2, arise at energies of 1.489, 1.508, and 1.518 eV, with full widths at half maximum (FWHM) of 5.5, 7.7, and 6.1 meV, respectively. They correlated peak 1 with the first QD level, peak I with the type-II interface, and peak 2 with the second QD level. They confirmed the labelling of the emission peaks with the PLE spectrum. A good agreement between measured data and calculations (using a one-band effective mass model in full 3D) was found, thus indicating a 0-dimensional DOS at the WZ–ZB GaAs interface. For the 3D calculations, they approximate the NW as a cylinder of WZ-GaAs (diameter of 16.6 nm) with a ZB-GaAs segment of 13 nm with 10 nm of AlAs coaxial with the NW. The vertical lines in Fig. 6a correspond to the calculated values. The calculations show two energy levels separated by 48 meV in the ZB QD, comparable with the measured value of 29 meV. The emission from the ZB QD and the interface when the excitation energy is varied (increasing from top to bottom, as indicated with arrows in the PLE spectrum) is shown in Fig. 6d. When the excitation energy increases, the emission from both the first QD level and the interface arises. The emission from the second QD level will appear only after the excitation energy surpasses the WZ-GaAs confined band gap (*E*_exc_ = 1.56 eV, purple line). At this point, the emission from the first QD energy level is strongly increased. State filling could be the reason behind this behaviour. Below this energy, the QD is excited resonantly, but charge carriers can diffuse from the WZ-GaAs barrier into the QD and recombine there. When the number of generated carriers is higher than the emitted through the first QD energy level, then the emission from the second QD level emerges. This behaviour is different from the one reported by Vainorius and co-workers^29^ for WZ–ZB-GaAs QWs. There the state filling leads to a shift in the type-II emission. They also showed that for the excitation energy of 1.61 eV (cyan line) the emission from the second QD level, and the emission from the interface, completely disappears. However, the mechanism behind this behaviour is still unclear.

### 3.2 Electronic properties

A constant quest for architectures where a quantum state, such as spin, can be effectively controlled has been sparked by emerging theoretical concepts for quantum technologies. QDs provide significant advantages over actual atoms in the manipulation of their properties using electric and magnetic fields. Compared to other systems,^163–169^ the crystal phase approach presented in this review allows size control in 3-D with atomically abrupt interfaces^22,28,135^ as presented in Sections 2 and 3.1. The size control, together with the strong spin–orbit interaction and a large *g*-factor observed for InAs^76^ and InSb,^170^ provides a cleaner system to study quantum phenomena not achievable in other systems.^163–169^ Associations of two quantum dots, in series or in parallel, are named double quantum dots (DQDs) or artificial molecules. DQDs allow the control of charge, spin, and orbital interactions and are important systems to study quantum phenomena. Parallel DQDs have been demonstrated in crystal phase InAs NWQDs, where the ZB-InAs QDs were sandwiched between WZ-InAs tunnel barriers.^171^ Thanks to the intrinsic characteristics of the crystal phase approach, they fabricated extremely thin (∼5 nm) ZB-InAs QDs. They employed three gate electrodes to tune the potential profile to either host one or two QDs. Finally, they demonstrated control over the interdot tunnel coupling and determined two coupling regimes, named weak (0.32 meV) and strong (3.1 meV) coupling. Moreover, single-electron transport has also been demonstrated in InAs NWQDs.^83,172^ In ref. 72, the length of the ZB-InAs QDs is correlated with the frequency of Coulomb oscillations to confirm the role as tunnel barriers of the WZ-InAs segments. They observed the Coulomb blockade effect over a broad range of gate voltages which provided an estimation of around +95 meV of the conduction-band offset between WZ- and ZB-InAs.

Due to the opportunities opened in the design, the electrical characteristics of crystal phase core–shell structures have been intensively investigated. For example, the InAs-GaSb core–shell system is particularly appropriate for devices such as tunnelling field effect transistors (TFETs) and diodes due to the type II (staggered) band alignment between InAs and GaSb.^173^ This material system has been extensively investigated in the axial and radial configurations.^146,173–176^ Namazi and co-workers explored the electrical properties of WZ core/shell InAs/GaSb NWs, where the WZ-GaSb is enabled by the crystal structure transfer method. They observed ambipolar behaviour at 4.2 K, which is indicative of parallel electron and hole channels. Moreover, they observed higher hole conductance in the WZ InAs/GaSb core/shell structure compared to the ZB-GaSb axial segment. This is indicative of a positive offset in the valence band edge of WZ compared to ZB in agreement with theoretical predictions.

#### 3.2.1 Quantum rings in ZB-InAs NWQDs

**Fig. 7:**
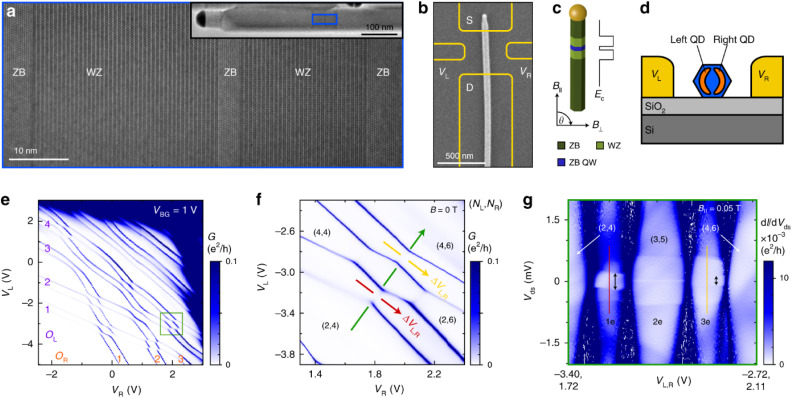
(a) Transmission electron micrograph of a representative nanowire. The centre ZB segment acts as a quantum well accessed by WZ tunnel barriers. (b) SEM of the studied nanowire, overlaid with the contact design. (c) Schematic of the crystal structure and resulting conduction-band alignment. (d) Side-view illustration of the formation of two parallel-coupled QDs. (e) Conductance *G* as a function of side-gate voltages (*V*_L_, *V*_R_), where the orbital numbers (*O*_L_, *O*_R_) are indicated. (f) Magnification of the (*O*_L_, *O*_R_)  =  (2, 3) crossing. Green, red, and yellow arrows indicate important gate vectors, and (*N*_L_, *N*_R_) represents the electron population on the left and right QD. (g) Measurement of d*I*/d*V*_ds_*versus V*_ds_ along the green gate vector for *B*_‖_ = 0.05 T. The Zeeman splitting of the ground state 2*E*_Z_ is indicated with arrows in the 1e and 3e regimes. From Fig. 1 by Potts, H., Chen, I., Tsintzis, A. *et al.*, Electrical control of spins and giant *g*-factors in ring-like coupled quantum dots, *Nat. Commun.*, **10**, 5740, (2019), https://doi.org/10.1038/s41467-019-13583-7. Is licensed under CC BY 4.0.

Potts and co-workers^76^ studied quantum rings that appear inside ZB-InAs crystal phase QWs. The InAs crystal phase QDs are shown in Fig. 7a, having a 5 nm ZB segment sandwiched between two WZ segments of about 30 nm that act as tunnel barriers due to the band alignment between WZ and ZB. They fabricated source-, drain- and gate-electrodes (Fig. 7b) to probe the electronic properties of the QW, and to study transport measurements in a dilution fridge equipped with a vector magnet.

The electrostatics in the QW were manipulated using two side gates (*V*_L_ and *V*_R_) and a back-gate (*V*_BG_). This way, two QDs, coupled to source and drain, form in the ZB-InAs segment (Fig. 7d). The DQD is proven by the honeycomb diagram observed in Fig. 7e. The strong confinement observed in the ZB-InAs is key in the electron population and occupancies of each QD and in the tunning of the inter-dot tunnel coupling thanks to the confinement provided by the crystal phase approach.^171^ The first orbital crossing (*O*_L,R_  =  1) was probed in previous work of the same research group.^171,177,178^ However, in ref. 76 they focused on the conductance maps of the interactions of higher orbitals. A magnification of the crossing (*O*_L_, *O*_R_)  =  (2, 3) (highlighted in Fig. 7e), together with the electron population, is shown in Fig. 7f. In conductance maps, for a standard DQD configuration, sharp corners are interpreted as no inter-dot tunnelling, as it can be observed for the (2,3) crossing. In contrast, rounded corners appear when there is a clear hybridization gap as in the (2,2) crossing.

The differential conductance, d*I*/d*V*_ds_, is represented in Fig. 7g*versus* the drain-source voltage (*V*_ds_) and the side-gate voltages (*V*_L,R_) for *B*_‖_ = 0.05 T aligned with the NWQD. Characteristic Coulomb diamonds are obtained in the dI/dV plot as a function of *V*_ds_ and *V*_L,R_ (Fig. 7g) for the 1, 2, and 3 electron regimes. The effective g*-factors obtained (∼64 and 40) were significantly larger than those for bulk InAs (−14.9).^179,180^

Moreover, they found interacting orbitals exhibiting an electronic structure almost identical to carbon nanotube (CNT) QDs (a quantum ring platform with a fourfold orbital- and spin-degeneracy, and anisotropic effective g*-factors), with highly anisotropic g*-factors varying from 3 to 80. However, in contrast to CNTs, it is possible to control the g*-factor from ∼80 to ∼0 using electric fields for the same charge state. Using perturbation theory, they confirmed that ring-like DQDs appear when one odd and one even orbital from each QD hybridize.

Quantum rings can also be employed to explore the Aharonov–Bohm (AB) effect as reported in 2022 by Debbarma and co-workers.^181^ The AB effect is a quantum mechanical phenomenon in which an electrically charged particle is affected by an electromagnetic potential, despite being confined to a region in which both the magnetic and electric fields are zero.^182^ In their work, they investigated two different kinds of crystal phase ZB-InAs (4–5 nm thick) ring-like QD structures (A and B). A pure ZB-InAs QD for sample A and the same structure together with a thin InAsSb shell for sample B. Surface band bending in the InAs and in the thin InAsSb shell is crucial for the appearance of the ring-like QD in InAs^183^ and InAsSb.^184^ The strong confinement inside the QD produced the absence of filled states, while electrons can accumulate close to the surface.^76^ However, the resulting ring-like states around the NW surface are sensitive to perturbations, resulting in localized electron states.^171^ They include two different samples to enhance the surface electron accumulation and to screen symmetry-breaking effects to further increase the spin–orbit interaction.

**Fig. 8:**
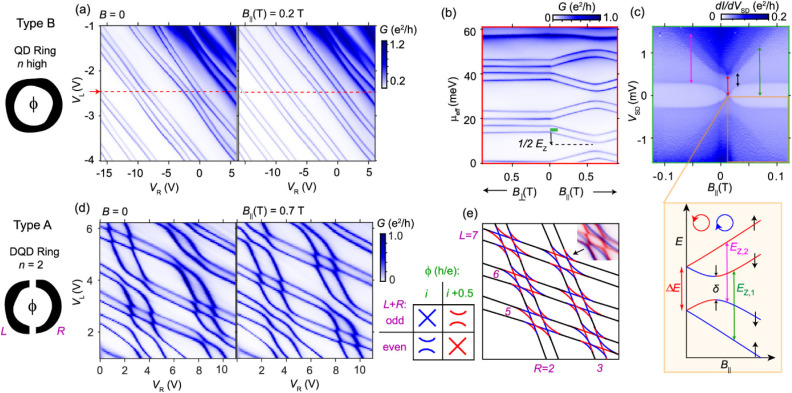
Effects of B-fields on orbital and spin states in QD rings with different symmetries (*n*). (a) Conductance of a QD ring (sample type B) as a function of *V*_L,R_ at *B* = 0 (left) and *B*_‖_ = 0.2 T (right) for a back-gate voltage, *V*_BG_ = 3 V, and source-drain voltage, *V*_SD_ = 0.1 mV. (b) Conductance along the red line in panel a *vs. B*_⊥_ and *B*_‖_. (c) Differential conductance *vs. B*_‖_ in the one-electron regime along the green bar in panel B. The inset shows schematically how the four nearly degenerate spin and orbital states evolve with *B*_‖_, where it is possible to identify *E*_Z_ and extract *g*_orb_ from *E*_Z_ = *g*_orb_*μ*_B_*B*_‖_. (d) Conductance of a double QD ring (sample type A) for *B* = 0 and *B*_‖_ = 0.7 T at *V*_BG_ = 0 V and *V*_SD_ = 0.5 mV. (e) Schematic for six consecutive orbital crossings at *ϕ* = 0 and 0.5 h/e. Orbital parity and threaded flux (*i*, integer) determine whether each crossing is exact or avoided. The small inset shows an overlay of data from panel d at the two fields. From Fig. 2 by Debbarma. R, *et al.*, *Nano Lett.*, 2022, **22**, 1, 334–339, https://doi.org/10.1021/acs.nanolett.1c03882 is licensed under CC BY 4.0.

Fig. 8 shows the conductance and differential conductance maps for both type A and type B ring-like QDs. The conductance map as a function of *V*_L,R_ of the type B sample does not show any honeycomb structure (Fig. 8a). This is an indication of transport through a single QD.^185^ They found that the level structure and magnetic field evolution (Fig. 8b) are similar to the observed for CNT QDs. From Fig. 8b, the authors extracted *g*_orb_ = 290 and an upper limit for |*g*_spin_| of approximately 15, results that are consistent with those extracted from Fig. 8c. Moving to the type A structure, the conductance map (Fig. 8d) is explained by transport through a DQD parallel-coupled to source and drain.^171,186^ The numbering of the orbital crossings is indicated in the schematic in Fig. 8e. The authors highlight that the DQD is not ideal and the gate coupling to some orbitals is not constant over the whole measurement interval. They conclude that thanks to the strong phase coherence of the system, uncommon for non-superconducting rings, they could study theoretical predictions on the effects of symmetry and parity of a quantum ring. In this case, the crystal phase approach opens the possibility to engineer new material and crystal phase combinations that could provide an even more efficient manipulation of electronic states in ring-like DQDs.

## 4. Conclusions

In this review article, we make a comprehensive revision of the field of crystal phase NWQDs. Traditionally NWQDs are formed *via* heterostructure interfaces between materials with different band gaps. However, their perfection is limited by the difficulty to control the interface abruptness and material composition at the atomic level, which unfortunately are determining factors for the physical properties of QDs as they define the shape of the potential well. Here, we discuss the growth of NWQDs in the axial direction varying the crystal phase, instead of the material composition, emphasizing the advantages offered by this approach. As this method employs a change in crystal structure instead of a change in material, then perfectly abrupt interfaces can be achieved. However, the band offset between WZ and ZB is fixed and relatively small, limiting the application of crystal phase QDs to liquid-He temperatures and to fields where the purity of the interface is more critical than the band offset. This is the case of the inspection of fundamental optical and quantum effects. We review the concepts of WZ and ZB crystal structures, supersaturation, and the different options available to control the crystal structure. For controlling the crystal phase, scientists have developed a method where only the group-V supply should be changed. In addition, different crystal phases offer different lateral facet terminations and, consequently, different surface energies, allowing to grow the shell in a selective fashion. That is, the shell can be grown either on the WZ or on the ZB phase of the core, thus leading to the formation of ring-like structures or enabling the growth of crystal phases that would be otherwise very difficult, if not impossible, to obtain. As an example, we describe the case of WZ-GaSb. Then, in Section 3, we discuss the optical and electronic properties of crystal phase NWQDs. The optical properties are focused on the type II transitions that appear in ZB-GaAs QDs and on the atomically sharp nature of the interface between WZ and ZB. Regarding the electronic properties, we mainly analyse the coupling between two QDs (forming DQDs) and the ring-like behaviour of ZB-InAs parallel-coupled DQDs. We strongly believe that crystal phase NWQDs are an excellent platform to discover new quantum phenomena either to probe theoretical models or to engineer new material architectures by means of the crystal structure transfer method.

## Conflicts of interest

There are no conflicts to declare.

## Supplementary Material
